# Severe Plastid Genome Size Reduction in a Mycoheterotrophic Orchid, *Danxiaorchis singchiana*, Reveals Heavy Gene Loss and Gene Relocations

**DOI:** 10.3390/plants9040521

**Published:** 2020-04-17

**Authors:** Shiou Yih Lee, Kaikai Meng, Haowei Wang, Renchao Zhou, Wenbo Liao, Fang Chen, Shouzhou Zhang, Qiang Fan

**Affiliations:** 1 Shenzhen Key Laboratory of Southern Subtropical Plant Diversity, Fairylake Botanical Garden, Shenzhen & Chinese Academy of Sciences, Shenzhen 518004, China; leesy3@mail.sysu.edu.cn; 2 State Key Laboratory of Biocontrol and Guangdong Provincial Key Laboratory of Plant Resources, School of Life Sciences, Sun Yat-sen University, Guangzhou 510275, China; mengkk@mail2.sysu.edu.cn (K.M.); lsswhw@foxmail.com (H.W.); zhrench@mail.sysu.edu.cn (R.Z.); lsslwb@mail.sysu.edu.cn (W.L.); 3 Administrative Committee of Danxiashan World Natural Heritage and Global Geopark, Shaoguan 521300, China; waynexiao800@gmail.com

**Keywords:** Calypsoinae, evolution, gene loss, nonphotosynthetic, plastome, pseudogene

## Abstract

*Danxiaorchis singchiana* (Orchidaceae) is a leafless mycoheterotrophic orchid in the subfamily Epidendroideae. We sequenced the complete plastome of *D. singchiana*. The plastome has a reduced size of 87,931 bp, which includes a pair of inverted repeat (IR) regions of 13,762 bp each that are separated by a large single copy (LSC) region of 42,575 bp and a small single copy (SSC) region of 17,831 bp. When compared to its sister taxa, *Cremastra appendiculata* and *Corallorhiza striata* var. *involuta*, *D. singchiana* showed an inverted gene block in the LSC and SSC regions. A total of 61 genes were predicted, including 21 tRNA, 4 rRNA, and 36 protein-coding genes. While most of the housekeeping genes were still intact and seem to be protein-coding, only four photosynthesis-related genes appeared presumably intact. The majority of the presumably intact protein-coding genes seem to have undergone purifying selection (d*N*/d*S* < 1), and only the *psa*C gene was positively selected (d*N*/d*S* > 1) when compared to that in *Cr. appendiculata*. Phylogenetic analysis of 26 complete plastome sequences from 24 species of the tribe Epidendreae had revealed that *D. singchiana* diverged after *Cr. appendiculata* and is sister to the genus *Corallorhiza* with strong bootstrap support (100%).

## 1. Introduction

Epidendroideae is the largest of the five subfamilies in the family Orchidaceae [1]. As this subfamily accounts for the majority of the orchid radiation and displays well-known orchid characteristics, the study of epidendroid diversification is deemed essential in orchid systematics. However, a portion of the members of this subfamily shows low photosynthetic ability and instead rely on mycoheterotrophy for nutrition [2]. As plants with mycoheterotrophic capabilities commonly have distinct anatomical, physiological, and genomic features, a reduction in plastid genome size is expected to occur due to changes in photosynthesis-related protein-coding genes, i.e., loss or pseudogenized [3]. Studies have revealed no significant changes in the plastome structure despite a reduction in the *ndh* complex [4]. Further investigations have proven that the evolution of heterotrophy occurs rapidly and independently across the genus [2,5]. Although studies on the plastome of selected mycoheterotrophic orchids have been performed by several plant researchers, the lack of comprehensive species samplings limits the amount of available information on the mycoheterotrophic lineages in these selected orchids. Therefore, additional information on mycoheterotrophic orchids of different genera and species is required to better understand the peculiar evolution that leads to plastome reduction.

To date, the complete plastome of a total of 30 species in 12 genera of mycoheterotrophic orchids in the family Orchidaceae has been reported. However, only *Cyrtosia septentrionalis* and *Rhizanthella gardneri* represent the subfamilies Vanilloideae and Orchidoideae [6,7], respectively, while the other species with a complete plastome belong to the Epidendroideae subfamily [5,8,9,10,11,12,13,14,15,16,17]. Although these orchids are reported as mycoheterotrophs, they can be categorized into two types, namely, photosynthetic mycoheterotrophs (or semi-mycoheterotrophs, not to be confused with partial mycoheterotrophs), which often have reduced leaves and/or photosynthetic pigmentation observed in deformed leaves or stem tissues, and full (nonphotosynthetic) mycoheterotrophs, which lack chlorophyll and are thus unable to produce carbohydrates through photosynthesis [17]. Studies on fully mycoheterotrophic orchid genera can provide unexpected yet valuable information about the evolutionary transition of nonphotosynthetic plants from partial mycoheterotrophy to full mycoheterotrophy [16].

The recently described mycoheterotrophic orchid genus, *Danxiaorchis* (Calypsoinae, Epidendreae) contains only two recorded species, which are full mycoheterotrophs and are restricted to specific habitats. The type species, *Danxiaorchis singchiana* J. W. Zhai, F. W. Xing, and Z. J. Liu., known as the Danxia orchid, is a distinct lineage with morphological features such as a labellum with a large Y-shaped callus and two sacs at the base, and cylindrical, fleshy seeds. The Danxia orchid is confined to Mount Danxia in Guangdong, China, after which the genus was named [18]. According to the most recent phylogenetic analysis of *Danxiaorchis*, the genus is sister to *Corallorhiza* and *Oreorchis*, although *Oreorchis* could be paraphyletic to the other two genera [19].

Research on orchids, the largest family of flowering plants [20], has moved from phylogenetic analyses using short gene sequences to those using genome-scale data. Researchers have sought to understand the extraordinary diversification and genetic evolution of these higher plant species, especially the species that evolved full mycoheterotrophy and leaflessness. In this study, we conducted sequencing, assembly and gene annotation of the *D*. *singchiana* plastome. Our aim was to reveal the extent of reductive plastome evolution in *D. singchiana*, to perform comparative analyses between *D. singchiana* plastome and published plastomes of selected photosynthetic and nonphotosynthetic sister genera in subtribe Calypsoinae, and to construct a phylogenomic tree in order to clarify the molecular placement of *D. singchiana* in tribe Epidendreae at the genome level.

## 2. Results

### 2.1. Plastome Size and Structure

**Figure 1:**
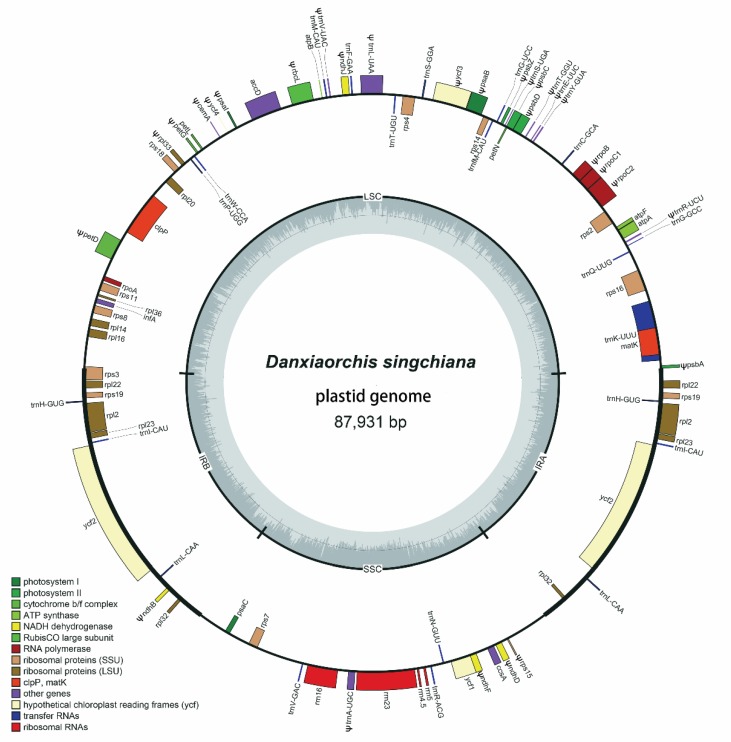
Plastid genome map of *Danxiaorchis singchiana*. Genes shown inside circles are transcribed in a clockwise direction, and those outside the circle are transcribed in a counterclockwise direction. Pseudogenes are indicated with ψ.The figure legend shows the gene functions based on color codes.

With next-generation sequencing, a total of 32,313,250 raw reads were obtained. Raw reads were directly fed into the pipeline without quality filtering or trimming in order to obtain the maximum amount of useful data. To speed up the assembly of plastid genomes, we chose only the first 2.5 million sequences in each paired-end dataset for downstream assembly. Finally, a total of 71,364 aligned reads were acquired, and 65,892 assembled reads were incorporated in the plastome assembly, with an average coverage depth of 122 times per site. The complete plastome of *D. singchiana* has a quadripartite structure and is 87,931 bp in length (Figure 1), containing a pair of inverted repeats (IRs; 13,762 bp each) that separate the large single-copy (LSC; 42,575 bp) region and the small single-copy (SSC; 17,831 bp) region. The total GC content of the plastome is 34.54%.

### 2.2. Gene Content and Order

**Figure 2:**
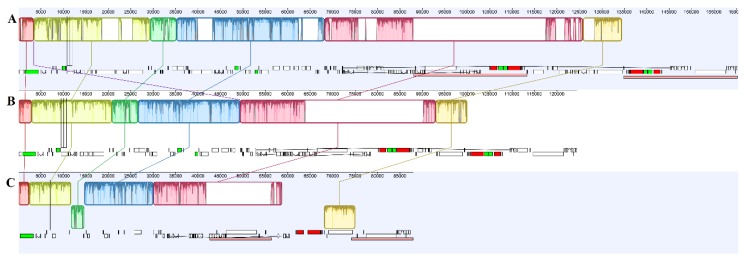
Plastome sequence alignment between (**A**) *Cresmastra appendiculata*, (**B**) *Corallorhiza striata* var. *involuta*, and (**C**) *Danxiaorchis singchiana*, using progressiveMAUVE [21].

For *D. singchiana*, we predicted a total of 61 genes, among which 36 were protein-coding genes, 21 were tRNA genes, and four were ribosomal RNA genes. However, nine of them were duplicates in the IR regions (Table 1). *Cremastra appendiculata* is the closest relative with a known plastome sequence available in the NCBI GenBank database. A comparison between the plastomes of *D. singchiana* and *Cr. appendiculata* revealed that 27 genes were pseudogenized, while 25 genes were lost (Table 1). Based on the classification scheme used in a previous study [5], the genes in the plastome were further grouped into five categories, namely, housekeeping, photosynthesis-related, NADPH dehydrogenase, plastid-encoded RNA polymerase, and ATP synthase. Among the housekeeping genes, 12 of 61 (19.7%) were recorded as pseudogenes or lost, with eight tRNA pseudogenes for the tRNA genes (*trn*A-UGC, *tr*nE-UUC, *trn*L-UAA, *trn*R-UCU, *trn*S-UGA, *trn*T-GGU, *trn*V-UAC, and *trn*Y-GUA) and one for the small subunit of the ribosome (*rps*15), while three tRNA genes (*trn*D-GUC, *trn*L-UAG, and *trn*S-GCU) were not found in the plastome. Among the photosynthesis-related genes, 28 of 31 (90.3%) were recorded as pseudogenes or lost, with the remaining presumably intact genes including the photosystem I subunit *psa*C, and the cytochrome *b_6_f* subunits *pe*tL and *pet*N. For the plastid-encoded RNA polymerase, only the subunit *rpo*A remained presumably intact. Interestingly, all the genes in the NADPH dehydrogenase (*ndh*), ATP synthase (*atp*), and photosystem II (*psb*) subunits of the photosynthesis-related genes were either pseudogenized or lost in *D. singchiana* plastome. By using progressiveMAUVE to detect the presence of large-scale evolutionary events among *Cr. appendiculata*, *Corallorhiza striata* var. *involuta*, and *D. singchiana*, we found two inversions relative to the conserved gene order in angiosperm plastomes in *D. singchiana* but none in *Co. striata* var. *involuta* (Figure 2). The inversions each occurred in the LSC (2.97-kb in length) and SSC (6.9 kb in length) regions, with the LSC inversion including only the protein-coded gene for *pet*N and pseudogenes *psb*C, *trn*E-UUC, *trn*S-UGA *trn*T-GGU, and *trn*Y-GUA and the SSC inversion consisting of the presumably intact genes *trn*N-GUU and *ycf*1 and pseudogenes for *ccs*A, *ndh*F, *ndh*D, and *rps*15.

### 2.3. Genome Comparison and Selection Pressure Analyses

When compared to the other 25 complete plastomes from tribe Epidendreae, the plastome of *D. singchiana* displayed the smallest total size (Table S1). More precisely, the lengths of its LSC region and IR region were the shortest across all species that were included in the study, but not that of its SSC region. The SSC region of *D. singchiana* is intermediate in length compared with the SSC region lengths of the other orchid species and shorter than the SSC region of *Anathalis obovata*, *Cr. appendiculata*, *Masdevallia coccinea,* and *Masdevallia picturata*. In the d*N*/d*S* analysis, the overall d*N*/d*S* for the concatenated dataset was less than 1.0, which was due to signals of purifying selection (Table S2). When analyzed separately, the 25 genes displayed purifying selection, whereby changes in amino acid residues in the genes that could favor an excess of synonymous versus nonsynonymous substitutions were prevented. The chloroplast gene *psa*C, displayed positive selection, whereby the protein sequences were altered, in *D. singchiana*, whereas the other four groups of genes did not exhibit any nonsynonymous (K_a_) or synonymous (K_s_) values indicative of selection due to the constraints of the used model.

### 2.4. Phylogenomic Analysis

**Figure 3:**
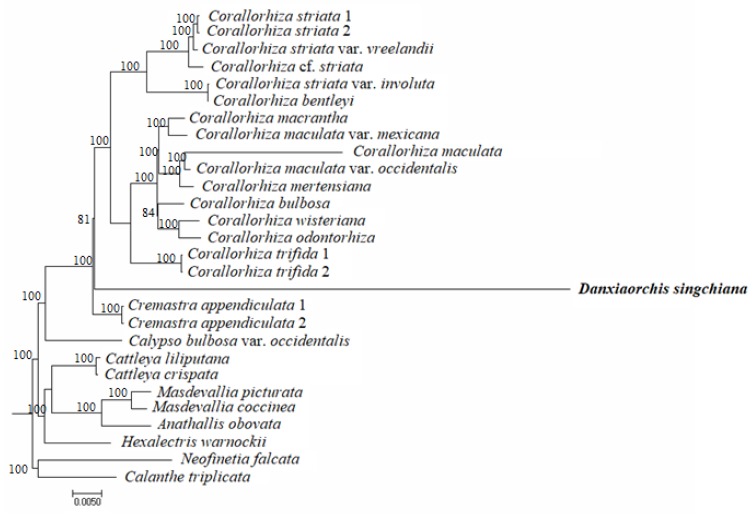
Maximum-likelihood (ML) tree of 26 complete plastome sequences derived from the 23 orchid species in tribe Epidendreae. The GenBank accession numbers for the orchid species used in this study are listed in Table S1. Two closely-related species, *Calanthe triplicata* (tribe Collabieae) and *Neofinetia falcata* (tribe Vandeae), were included as outgroups.

The maximum-likelihood (ML) tree was based on the complete plastome sequences of 26 orchid species in tribe Epidendreae and demonstrated that *D. singchiana* diverged after *Cr. appendiculata* and was sister to the genus *Corallorhiza* (Figure 3). This molecular placement was supported by strong bootstrap values at almost all branch nodes.

## 3. Discussion

In general, land plants contain a chloroplast (cp) genome between 120 and 160 kb in size, while differences in cp genome size are mostly influenced by the expansion/contraction of IR regions due to changes in the amount of repeated DNA and/or changes in sequence complexity [22]. This is true for *D. singchiana* as the IR sequence length was halved compared to the IR sequence length in the other species of the same subtribe (Table S1). However, its SSC sequence length was slightly larger than that of the other mycoheterotrophic orchid species in the same subtribe but shorter than that of other photosynthetic orchid species in the same tribe. *Cremastra appendiculata*, which is the closest relative of *D. singchiana* with a known plastome, contains 129 genes, including 86 protein-coding, 35 tRNA, and 8 rRNA genes. Upon comparison, we found that 50, 14, and four protein-coding, tRNA, and rRNA genes, respectively, were lost in *D. singchiana.* As *D. singchiana* diverged after *Cr. appendiculata*, it experienced a 52.7% reduction in its total gene abundance. The major loss of presumably intact genes mainly occurred for the photosynthesis-related, *ndh*, plastid-encoded RNA polymerase, and *atp* genes, while the amount of gene loss was minimal among the housekeeping genes. In our study, the presence of pseudogenes was based on gene predictions computed with the gene annotation tool GeSeq, whereby precise annotation was based on the similarity of the protein sequence with a proper start codon [23]. The plastome sequence of *D. singchiana* was aligned against the plastome sequence of *Cr. appendiculata* and crosschecked for possible pseudogenized gene sequences and confirmed gene losses. On the basis of our observation, pseudogenes are present among protein-coding loci in the plastome for three reasons that cause them to be putatively nonintact: (1) premature stop codons within the gene sequence, (2) frequent deletion of gene sequences at both ends, and (3) the occurrence of unexpected single nucleotide polymorphisms (SNPs) in the start codon that result in its loss.

Losses of *ndh* genes in mycoheterotrophic orchids are common, as demonstrated in several genera in family Orchidaceae and in Calypsoinae, and consistent *ndh* gene loss was detected in the genus *Corallorhiza* [5] (Figure S1). It was proven that some photoautotrophic and all heterotrophic plants lack a functional *ndh* complex and thus, are likely to be missing plastid-encoded *ndh* genes [24]. Furthermore, multiple independent degradation pathways may exist in the orchid family according to the presence of variations in the status of *ndh* genes among the orchid plastomes that have been reported thus far [4]. The literature had shown that in some plant species, however, *ndh* genes have been transferred to the mitochondrial genome, usually in a truncated form or with large deletions [25]. The loss of *ndh* genes is strongly correlated with the instability of the SSC/IR junction in Orchidaceae, in which the species with presumably intact *ndh*F genes exhibit a part of the *ycf*1 sequence (approximately 1 kb) located within the IR, while the *ndh*F gene is located very near to the other end of the SSC/IRA junction [7]. For species with *ndh*F gene deletions, the *ycf*1 gene was observed to have varying sequence lengths over the SSC/IRA junction, to be completely placed within the IR region, or to be completely in the SSC region but with its sequence end less than 21 bp away from the SSC/IRA junction [25]. However, in *D. singchiana*, we observed that the deletion of *ndh* genes severely shifted the IR boundary, resulting in extensive structural rearrangement whereby the *ycf*1 gene was completely placed within the SSC region, which was 3,940 bp away from the SSC/IRB junction. On the other hand, the pseudogenized *ndh*F gene was found to be located at the 3′ end, before the *ycf*1 gene (Figure 1). This unique translocation of the *ycf*1 gene was also accompanied by the 6.9-kb gene block inversion detected in the SSC region (Figure 2). As inversions of the LSC often correspond to sequence alterations in IR regions [26], the occurrence of similar events could be proposed for the SSC region of *D. singchiana*. Genetic inversions are usually found in plastomes of parasitic and mycoheterotrophic plants but rarely in photosynthetic plants [4,7,12,27,28]. Inversions in plastomes could result from homologous recombination between repeated sequences and could lead to rearrangement of gene order [29]. In this study, the two inversions that were detected in the *D. singchiana* plastome were found to be unique to the species. Such a phenomenon might lead to unexpected findings, and the mechanism of the natural development of genetic inversions in the species could be related to its unique lifestyle. Nevertheless, in-depth studies are needed to obtain a better understanding of this phenomenon.

Because of the absence of almost all photosynthesis-related genes in the plastome, the loss-of-function in plastid-encoded *atp* genes is expected. Even so, most nonphotosynthetic orchid species that experienced a substantial loss of photosynthesis-related genes still retain the *atp* complex, such as *Corallorhiza* spp. (Figure S1). The *atp* complex is responsible for ATP synthesis in photosynthesis, also called photosynthetic phosphorylation or photophosphorylation [30]. Cyclic photophosphorylation requires only a light source for complete photosynthesis and is driven by photosystem I, while noncyclic photophosphorylation is linked to oxygen evolution, water oxidation, and acceptor reduction [31] and requires both photosystem I and II [32]. As a result of few to no photosynthesis-related genes being found in the *D. singchiana* plastome, the *atp* complex was reduced and experienced gene loss. On the other hand, the retention of the *atp* complex in *Corallorhiza* could be regulated by an unknown, photosynthesis-independent mechanism. Such a mechanism was proposed to function in maintaining a proton gradient between the thylakoid lumen and stroma, which is a criterion for the Tat-dependent protein translocation system [33].

Reductive evolution could be a consequence of a nonphotosynthetic lifestyle [34], in which the mycoheterotrophic plant relies on the microbiome community in its rhizome for consistent nutrient uptake. Although the plastome of *D. singchiana* experienced extensive gene loss, the conservation of essential plastid genes plays an indispensable role in cellular viability and thus ensures the plant’s survival [35]. In a comprehensive review, a total of 52 genes were identified to be essential to heterotrophic growth [36]. Among these genes, nine were lost from the *D. singchiana* plastome, namely, *trn*A-UGC, *trn*D-GUC, *trn*E-UUC, *trnL*-UAA, t*rnL*-UAG, *trn*R-UCU, *trn*S-GCU, *trn*V-UAC, and *trn*Y-GUA, which were all in the transfer RNA gene family. At least 25 transfer RNA genes are required for a plant plastome to be functional [37]. A study predicted that the *trn*E gene, which is essential for haem biosynthesis [38], will cling to the plastome even when plant cell lines experience plastome deletion [39]. However, the *trn*E gene is predicted to be a pseudogene in the *D. singchiana* plastome. The ribosomal RNA genes, on the other hand, were still all presumably intact in the *D. singchiana* plastome. This suggests that the plastome in *D. singchiana* remains functional; however, the essential transfer RNA genes were incomplete. Therefore, translation of *D. singchiana* plastid mRNAs is probably to involve tRNAs of cytosolic origin [40], suggesting that tRNA import may occur in *D. singchiana* plastome to warrant its functionality.

The first phylogenetic analysis of *D. singchiana* was performed against 71 genera of Orchidaceae using a combined dataset of three gene/loci, including the two protein-coding plastid genes *mat*K, and *rbc*L and the nuclear ribosomal DNA internal transcribed spacer (ITS) region [18]. Although the finding revealed that *Danxiaorchis* is a sister species of *Yoania*, the inclusion of the partial *rbc*L gene sequence of *Yoania japonica* in the phylogenetic analysis was questionable. Thus, a comprehensive phylogenetic analysis of 37 species in 14 genera of subtribe Calypsoinae was carried out using the combined *mat*K and ITS dataset [19]. The new phylogenetic tree revealed that *D. singchiana* is a sister species to the *Oreorchis*+*Corallorhiza* clade under a strong Bayesian posterior probability (0.90) and moderate ML bootstrap support (77%). The same analysis criteria were also used when reporting the molecular placement of *Danxiaorchis yangii*, a novel congener that was discovered a few years after *D. singchiana*, on Mount Jinggang of Hunan province, China [41]. Finally, the results of the phylogenetic analysis carried out using the complete genome sequence of 26 species in tribe Epidendreae were congruent with the phylogenetic relationships in subtribe Calypsoinae proposed in a previous study [19]. Although a minor inconsistency in molecular placement within the *Corallorhiza* clade was observed, we concluded that the ML tree constructed using the complete plastome sequences was capable of depicting the phylogenetic relationships among orchid species in subtribe Calypsoinae and is, therefore, useful at the subfamily level.

Identifying a suitable gene region for phylogenetic analysis in Orchidaceae is rather challenging due to gene absence and pseudogenes in some orchid species. In fact, pseudogenes are not favorable in phylogenetic analysis in some orchid species [42]. As a result, the application of *mat*K and ITS in the construction of a phylogenetic tree for Epidendreae is promising [19]; *rbc*L is a pseudogene in mycoheterotrophic orchids, such as *D. singchiana* and *Y. japonica*. However, the *mat*k sequence was not found in *Yoania amagiensis* based on a dataset of NGS sequences [19]. The absence of the *mat*K gene in *Y amagiensis* may be individual-specific, as different gene numbers were detected in the same mycoheterotrophic/photosynthetic orchid, e.g., *Co. striata* and *Cr. appendiculata*. We suggest performing additional NGS sequencing to clarify the presence of the *mat*K gene in *Y. amagiensis* in order to provide better resolution for mycoheterotrophic orchids in Epidendreae.

Mount Danxia, from which *D. singchiana* originated, is speculated to have formed 6 million years ago (Mya) [43]. Researchers have suggested that the unique geological conditions and relative environmental isolation of this area give rise to rare and endemic species with restricted habitats [44]. The presence of *D. singchiana* could be a good example of genetic isolation and speciation leading to the evolution of the mycoheterotrophic lifestyle due to its distinctive habitat. To date, there is limited information on the molecular dating of *D. singchiana*, which is based on three mitochondrial protein-coding genes, namely, *atp*1, *mat*R, and *nad*5 [45]. The divergence time between *Cr. appendiculata* and *D. singchiana* was speculated to have been approximately 11.06 Mya. To clarify the relationship between the formation of Mount Danxia and the speciation of *D. singchiana*, sequencing of its only congener, *D. yangii*, could provide better insights into the evolution traits of this mycoheterotrophic species and aid in deciphering the plastome evolution in *Danxiaorchis* as a whole.

## 4. Materials and Methods

Fresh aboveground tissue of *D. singchiana* was collected from Mount Danxia, Guangdong Province, China. Genomic DNA was extracted using a modified CTAB protocol [46]. A genomic library with an insert size of ~350 bp was prepared using a TruSeq DNA Sample Prep Kit (Illumina, USA) and sequenced on an Illumina NovaSeq platform (Illumina, USA) to obtain pair-end reads of 150 bp. Upon removal of adapter sequences using NGSToolkit [47], the raw reads were subjected to *de novo* assembly using NOVOPlasty 2.7.2 [48], in which the *rbc*L gene sequence of *D. singchiana* (Genbank accession: JX293187) was used as the initial seed. A single contig was obtained at the end of the assembly process. The assembled genome was initially annotated and its junction points between the LSC, SSC, and IRs were identified using the online annotation tool GeSeq [23]; then, the genome was manually checked for annotation errors. The plastome sequence was deposited in NCBI GenBank under accession number MN584923. Plastome alignment was conducted using the web-based alignment software MAFFT [49] with 26 complete plastomes of 23 species in the Epidendreae tribe downloaded from NCBI GenBank (Table S1), and the locations of pseudogenes were manually identified by crosschecking the unannotated gene sequences. The size of the plastome was compared with that of the other 23 species, and a circular plastome map was visualized in OGDRAW 1.3.1 [50].

Based on recent phylogenetic analysis and classification of the Calypsoinae subtribe, *Cremastra* is believed to be the closest genus to *Danxiaorchis*, and *Corallorhiza* and *Oreorchis* diverged after *Danxiaorchis* [19]. Subsequently, plastome comparative analysis was conducted for *D. singchiana* against *Cr. appendiculata* and *Co. striata* var *involuta*, whereby the plastomes were aligned using progressiveMAUVE [21], and the presence of large-scale evolutionary events such as rearrangement and inversion between *D. singchiana* and its sister genera was determined.

The ratio of nonsynonymous to synonymous substitutions (d*N*/d*S*) of protein-coding regions was calculated for *Cr. appendiculata* and *D. singchiana* (Table S1). Plastome alignment was conducted using MAFFT [49], and protein-coding genes present in both species were included in the analysis, using PAML 4.7 [51] to estimate the selection pressure acting on these genes. The Yang and Nielsen codon frequency model (yn00) was selected, with the parameters for the initial ratio of transitions to transversion frequency (Κ) set at 0.3 < K < 0.7. Calculations were carried out in two ways: (1) with a concatenated dataset containing 30 protein-coding genes and (2) with all 30 protein-coding genes analyzed separately.

For phylogenomic tree construction, the plastome sequence of *D. singchiana* and 25 complete plastomes of 23 species in tribe Epidendreae were included. Two species, *Neofinetia falcata* (tribe Vandeae) and *Calanthe triplicata* (tribe Collabieae) were included as outgroups (Table S1). Plastomes were aligned using MAFFT [49] and trimmed using trimAl v1.2 [52] with the gappyout method in order to reduce the systematic errors produced by poor alignment, while an ML analysis was conducted using RAxML [53] available in the CIPRESS Science Gateway web portal (https://www.phylo.org/portal2/login!input.action) [54], using the general time-reversible (GTR) with gamma distribution (+G) (=GTR+G) nucleotide substitution model and 1000 bootstrap replicates for each branch node.

## Figures and Tables

**Table 1:** List of presumably intact genes in the plastome of *Danxiaorchis singchiana* when compared to that of *Cremastra appendiculata* (MH356724). Genes that were duplicated in the inverted repeats (IR) regions are marked with the symbol *.

Type of Genes	Group of Gene	Gene Name
Housekeeping	Large subunit of ribosome	*rpl*2*; *rpl*14; *rpl*16; *rpl*20; *rpl*22*; *rpl*23*; *rpl*32*; *rpl*33; *rpl*36
	Small subunit of ribosome	*rps*2; *rps*3*; *rps*4; *rps*7; *rps*8; *rps*11; *rps*12; *rps*14; *rps*16; *rps*18; *rps*19*
	Ribosomal RNA genes	*rrn*4.5; *rrn*5; *rrn*16; *rrn*23
	Transfer RNA genes	*trn*C-GCA; *trn*F-GAA; *trn*fM-CAU; *trn*G-GCC; *trn*G-UCC; *trn*H-GUG*; *trn*I-CAU*; *trn*I-GAU; *trn*K-UUU; *trn*L-CAA*; *trn*M-CAU; *trn*N-GUU; *trn*P-UGG; *trn*Q-UUG; *trn*R-ACG; *trn*S-GGA; *trn*T-UGU; *trn*V-GAC; *trn*W-CCA
	Translational initiation factor	*inf*A
	Maturase	*mat*K
	Subunit of acetyl-CoA carboxylase	*acc*D
	Subunit of protease Clp	*clp*P
	Component of TIC complex	*ycf*1; *ycf*2*
Photosynthesis-related	Subunits of photosystem I	*psa*C
	Subunits of cytochrome *b_6_f*	*pet*L; *pet*N
Plastid-encoded RNA polymerase	Subunits of the plastid-encoded RNA polymerase	*rpo*A

## Supplementary Materials

Supplementary materials can be found at https://www.mdpi.com/2223-7747/9/4/521/s1. Table S1: General information, GenBank accession numbers and plastome sizes of orchid species used in this study; Table S2: List of protein-coding genes used in the calculation of nonsynonymous to synonymous substitution ratio (d*N*/d*S* or K_a_/K_s_); Figure S1: Gene content heatmap of the 111 selected genes of 26 complete plastome sequences derived from the 23 orchid species under the tribe Epidendreae. All authors have read and agreed to the published version of the manuscript.
