# Supplementary material for: Severe Plastid Genome Size Reduction in a Mycoheterotrophic Orchid, Danxiaorchis singchiana, Reveals Heavy Gene Loss and Gene Relocations
Source: Plants (Basel). 2020 Apr 17;9(4):521. doi: 10.3390/plants9040521 (PMC7238169; doi:10.3390/plants9040521)
Supplement: Supplementary file 1 [file plants-09-00521-s001.zip › Figure S1.docx]

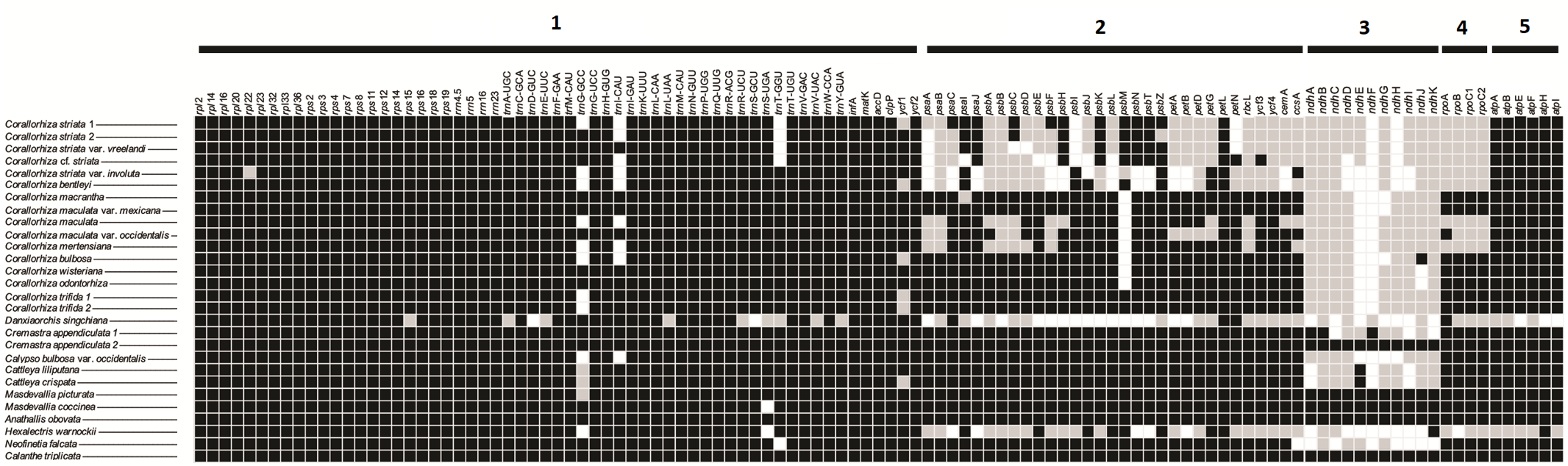


**Figure S1.** Gene content heatmap of the 111 selected genes of 26 complete plastome sequences derived from the 23 orchid species under the tribe Epidendreae. The GenBank accession numbers for the orchid species used in this study were listed in Table S1. For the gene content, the black-colored boxes represent presumably intact genes; the grey-colored and the white-colored boxes that indicates the pseudogenes and absent genes, respectively. Note: 1-housekeeping genes, 2-photosynthesis-related genes, 3-NADPH dehydrogenase, 4-plastid-encoded RNA polymerase, 5-ATP synthases.
