# Supplementary material for: Severe Plastid Genome Size Reduction in a Mycoheterotrophic Orchid, Danxiaorchis singchiana, Reveals Heavy Gene Loss and Gene Relocations
Source: Plants (Basel). 2020 Apr 17;9(4):521. doi: 10.3390/plants9040521 (PMC7238169; doi:10.3390/plants9040521)
Supplement: Supplementary file 1 [file plants-09-00521-s001.zip › Table S1.docx]

**Table S1** General information, GenBank accession numbers and plastome sizes of orchid species used in this study.

|  | **Tribe** | **Sub-tribe** | **Species^c^** | **GenBank accession number** | **Plastome size (bp)** | **Large single copy (bp)** | **Small single copy (bp)** | **Inverted repeat region (bp)** |
| --- | --- | --- | --- | --- | --- | --- | --- | --- |
| 1 | Epidendreae^a,c,d^ | Calypsoinae | *Calypso bulbosa* var*. occidentalis* | MG874037 | 149,313 | 84,543 | 14,846 | 24,962 |
| 2 |  |  | *Corallorhiza bentleyi* | MG874035 | 124,482 | 64,420 | 10,722 | 24,670 |
| 3 |  |  | *Corallorhiza bulbosa* | KM390018 | 148,643 | 83,422 | 15,343 | 24,939 |
| 4 |  |  | *Corallorhiza* cf*. striata* | MG874040 | 137,068 | 75,227 | 11,185 | 25,328 |
| 5 |  |  | *Corallorhiza macrantha* | KM390017 | 151,031 | 84,262 | 12,545 | 27,112 |
| 6 |  |  | *Corallorhiza maculata* var*. mexicana* | KM390015 | 151,506 | 84,347 | 12,671 | 27,244 |
| 7 |  |  | *Corallorhiza maculata* | KM390014 | 146886 | 80,401 | 12,885 | 26,800 |
| 8 |  |  | *Corallorhiza maculata* var*. occidentalis* | KM390016 | 146,595 | 81,362 | 12,369 | 24,362 |
| 9 |  |  | *Corallorhiza mertensiana* | KM390018 | 147,941 | 81,109 | 13,774 | 26,529 |
| 10 |  |  | *Corallorhiza odontorhiza* | KM390021 | 147,317 | 82,259 | 13,508 | 25,775 |
| 11 |  |  | *Corallorhiza striata* 1 | MG874034 | 141,202 | 75,701 | 13,319 | 26,091 |
| 12 |  |  | *Corallorhiza striata* 2 | MG874039 | 141,915 | 77,017 | 14,158 | 25,370 |
| 13 |  |  | *Corallorhiza striata* var*. involuta*^b^ | MG874038 | 124,433 | 63,344 | 10,189 | 25,450 |
| 14 |  |  | *Corallorhiza striata* var*. vreelandii* | JX087681 | 137,505 | 72,151 | 12,388 | 26,483 |
| 15 |  |  | *Corallorhiza trifida* 1 | MH874036 | 149,376 | 83,685 | 15,285 | 25,203 |
| 16 |  |  | *Corallorhiza trifida* 2 | KM390019 | 149,384 | 82,724 | 15,006 | 25,827 |
| 17 |  |  | *Corallorhiza wisteriana* | KM390020 | 146,437 | 82,350 | 11,743 | 26,172 |
| 18 |  |  | *Cremastra appendiculata* 1 | MG925366 | 155,320 | 87,098 | 15,478 | 26,372 |
| 19 |  |  | *Cremastra appendiculata* 2^b,c^ | MH356724 | 160,494 | 88,249 | 21,457 | 25,394 |
| 20 |  |  | *Danxiaorchis singchiana*^b,c^ | MN584923 | 87,931 | 42,575 | 17,832 | 13,762 |
| 21 |  | Laeliinae | *Cattleya crispata* | KP168671 | 148,343 | 86,254 | 13,261 | 24,614 |
| 22 |  |  | *Cattleya liliputana* | KP202881 | 147,092 | 85,804 | 13,900 | 23,694 |
| 23 |  | Pleurothallidinae | *Anathallis obovata* | MH979332 | 155,515 | 83,694 | 20,047 | 25,542 |
| 24 |  |  | *Masdevallia coccinea* | KP205432 | 157,423 | 84,957 | 18,448 | 27,009 |
| 25 |  |  | *Masdevallia picturata* | KJ566305 | 156,045 | 85,145 | 20,742 | 25,079 |
| 26 |  | Bletiinae | *Hexalectris warnockii* | MH444822 | 119,057 | 66,903 | 17,490 | 17,332 |
| 27 | Vandeae^d^ | Aeridinae | *Neofinetia falcata* | KT726909 |  |  |  |  |
| 28 | Collabieae^d^ |  | *Calanthe triplicata* | KF753635 |  |  |  |  |

Note: a-data used in plastome size comparison analysis; b-data used in progressiveMAUVE and Figure 2; c-data used in d*N*/d*S* analysis; d-data used in ML tree, gene content summary and Figure S1.
