# Supplementary material for: Severe Plastid Genome Size Reduction in a Mycoheterotrophic Orchid, Danxiaorchis singchiana, Reveals Heavy Gene Loss and Gene Relocations
Source: Plants (Basel). 2020 Apr 17;9(4):521. doi: 10.3390/plants9040521 (PMC7238169; doi:10.3390/plants9040521)
Supplement: Supplementary file 1 [file plants-09-00521-s001.zip › Table S2.docx]

**Table S2** List of protein-coding genes used in the calculation of nonsynonymous to synonymous substitution ratio (d*N*/d*S* or K_a_/K_s_).

| **No.** | **Gene** | **d*N* (K_a_)** | **d*S* (K_s_)** | **d*N*/d*S* (K_a_/K_s_)** | ***p*-value** | **Aligned sequence length (bp)** | **Substitutions** | | |
| --- | --- | --- | --- | --- | --- | --- | --- | --- | --- |
|  |  |  |  |  |  |  | Total | d*N* (K_a_) | d*S* (K_s_) |
| 1 | *acc*D | 0.019023 | 0.062114 | 0.306265 | 0.0007 | 1446 | 38 | 22 | 16 |
| 2 | *clp*P | 0.019788 | 0.048576 | 0.407348 | 0.069414 | 612 | 16 | 9 | 7 |
| 3 | *inf*A | 0.016983 | 0.10378 | 0.163643 | 0.015727 | 231 | 8 | 3 | 5 |
| 4 | *mat*K | 0.023337 | 0.040274 | 0.579459 | 0.122243 | 1338 | 36 | 23 | 13 |
| 5 | *pet*L | 0.032637 | 0.150158 | 0.21735 | 0.061244 | 93 | 5 | 2.3 | 2.7 |
| 6 | *pet*N | 0.015071 | NA | NA | NA | 87 | 1 | 1 | NA |
| 7 | *psa*C | 0.026346 | 0.020691 | 1.2733 | 0.745092 | 243 | 6 | 5 | 1 |
| 8 | *rpl*2 | 0.003267 | 0.046539 | 0.070201 | 9.53E-05 | 813 | 11 | 2 | 9 |
| 9 | *rpl*14 | 0.006992 | 0.039381 | 0.177544 | 0.068019 | 366 | 5 | 2 | 3 |
| 10 | *rpl*16 | 0.028642 | 0.036631 | 0.781902 | 0.659946 | 405 | 12 | 9 | 3 |
| 11 | *rpl*20 | 0.007243 | 0.038732 | 0.187 | 0.074859 | 357 | 5 | 2 | 3 |
| 12 | *rpl*22 | 0.017138 | 0.030198 | 0.567504 | 0.385306 | 363 | 7 | 5 | 2 |
| 13 | *rpl*23 | 0.009537 | 0.036765 | 0.259399 | 0.191572 | 267 | 4 | 2 | 2 |
| 14 | *rpl*32 | 0.038233 | NA | NA | NA | 162 | 5 | 5 | NA |
| 15 | *rpl*33 | 0.006192 | 0.120856 | 0.051235 | 0.003888 | 198 | 5 | 1 | 4 |
| 16 | *rpl*36 | NA | NA | NA | NA | 111 | 0 | NA | NA |
| 17 | *rpo*A | 2.13919 | 2.14065 | 0.999317 | 1 | 624 | 441 | 355.5 | 85.5 |
| 18 | *rps*2 | 0.027567 | 0.040582 | 0.679279 | 0.415071 | 708 | 21 | 15 | 6 |
| 19 | *rps*3 | 0.029483 | 0.062511 | 0.471648 | 0.105648 | 654 | 23 | 15 | 8 |
| 20 | *rps*4 | 0.012933 | 0.022752 | 0.568448 | 0.32077 | 603 | 9 | 6 | 3 |
| 21 | *rps*7 | 0.014221 | 0.064147 | 0.221697 | 0.01264 | 465 | 11 | 5.2 | 5.8 |
| 22 | *rps*8 | 0.023567 | 0.097089 | 0.242732 | 0.008624 | 393 | 15 | 7 | 8 |
| 23 | *rps*11 | 0.018069 | 0.068143 | 0.265157 | 0.035459 | 414 | 11 | 6 | 5 |
| 24 | *rps*12 | 0.017932 | 0.011629 | 1.54194 | 0.802147 | 369 | 6 | 5 | 1 |
| 25 | *rps*14 | 0.017191 | 0.031599 | 0.54403 | 0.38281 | 300 | 6 | 4 | 2 |
| 26 | *rps*16 | 0.021738 | NA | NA | NA | 228 | 4 | 4 | NA |
| 27 | *rps*18 | 0.02533 | 0.033022 | 0.767075 | 0.511389 | 303 | 8 | 6 | 2 |
| 28 | *rps*19 | 0.004658 | 0.033723 | 0.138128 | 0.112388 | 276 | 3 | 1 | 2 |
| 29 | *ycf*1 | 0.138446 | 0.154426 | 0.896521 | 0.354284 | 5217 | 668 | 542 | 126 |
| 30 | *ycf*2 | 0.014088 | 0.015478 | 0.910167 | 0.654192 | 6768 | 97 | 67 | 30 |
| 31 | Concatenated dataset | 0.960837 | 1.19091 | 0.806808 | 2.14E-116 | 24504 | 17648 | 14070.5 | 3577.5 |

Note: NA - no data available
